# Mimetic desire in autism spectrum disorder

**DOI:** 10.1186/s13229-016-0107-7

**Published:** 2016-11-08

**Authors:** Baudouin Forgeot d’Arc, Fabien Vinckier, Maël Lebreton, Isabelle Soulières, Laurent Mottron, Mathias Pessiglione

**Affiliations:** 1 Centre de Recherche du Centre Intégré Universitaire du Nord-de-l’Île-de-Montréal, Montréal, Canada; 2 Département de psychiatrie, Université de Montréal, Montréal, Canada; 3 Motivation, Brain and Behavior Team, Institut du Cerveau et de la Moelle épinière, Hôpital Pitié-Salpêtrière, 75013 Paris, France; 4 Service de Psychiatrie, Faculté de Médecine Paris Descartes, Centre Hospitalier Sainte-Anne, Université Paris Descartes, Sorbonne Paris Cité, Paris, France; 5 Psychology Department, Université du Québec à Montréal, C.P. 8888 succursale Centre-ville, Montréal, H3C 3P8 Canada; 6 Institut National de la Santé et de la Recherche Médicale, Centre National de la Recherche Scientifique, Université Pierre et Marie Curie, 75005 Paris, France; 7 ASD Specialized Clinic, Hôpital Rivière-des-Prairies, 7070 Blvd. Perras, Montréal, QC H1E 1A4 Canada

**Keywords:** Mimetic desire, Autism, Social influence, Social cognition, Social motivation, Mirror neuron system, Brain valuation system, Restricted interests

## Abstract

Mimetic desire (MD), the spontaneous propensity to pursue goals that others pursue, is a case of social influence that is believed to shape preferences. Autism spectrum disorder (ASD) is defined by both atypical interests and altered social interaction. We investigated whether MD is lower in adults with ASD compared to typically developed adults and whether MD correlates with social anhedonia and social judgment, two aspects of atypical social functioning in autism. Contrary to our hypotheses, MD was similarly present in both ASD and control groups. Anhedonia and social judgment differed between the ASD and control groups but did not correlate with MD. These results extend previous findings by suggesting that basic mechanisms of social influence are preserved in autism. The finding of intact MD in ASD stands against the intuitive idea that atypical interests stem from reduced social influence and indirectly favors the possibility that special interests might be selected for their intrinsic properties.

## Introduction

Reciprocal influence is an essential aspect of social behavior: individuals are influenced by others in their beliefs and preferences [1]. An essential element of this influence is mimetic desire (MD), which is the tendency to pursue goals pursued by others [16]. As an example, children often run after the same toy, even if other identical toys are available. MD is crucial for non-verbally sharing information about values (i.e., whether objects present in the environment are good or bad) without wasting time on trial-and-error learning and might therefore shape preferences during development. Two lines of reasoning led us to hypothesize that MD may be dysfunctional in autism spectrum disorder (ASD).

A first line of reasoning relates to clinical descriptions and cognitive investigations. Clinically, ASD is characterized by “deficits in social communication and interaction” and “restricted, repetitive behavior, interests or activities” [2]. It is also associated with altered social cognition [15] and atypical social motivation [11] including social anhedonia [9]. A lack of MD might underpin lower social influence on perceptual [8] and esthetic [11] judgments as well as learning [21] and donation decisions [20] associated with autism. An absence of MD would also compromise the sharing of desires and, hence, result in altered social interaction and possibly idiosyncratic preferences and atypical interests.

Another line of reasoning comes from neuroscience research. A recent study has empirically demonstrated MD in adults of the general population and revealed its neural basis [22]: visual objects are rated as more desirable once perceived as the goals of another agent’s action. According to this study, MD might result from a modulation of the brain valuation system (BVS) by the mirror neuron system (MNS), since MNS–BVS functional connectivity predicts individual susceptibility toward mimetic desires. In line with disconnection theories of autism [17], this functional connectivity between MNS and BVS may be altered in autistic individuals, such that others’ behavior would not affect their motivational system.

The present study aimed to assess whether MD is affected in autism, by testing the hypothesis that MD is (1) reduced in individuals with ASD relative to matched controls and/or (2) related to atypical social motivation and social cognition associated with ASD.

## Methods

### Participants

A power analysis using the “power.t.test” formula in the R package “stat” [26] was based on reported MD amplitude (mean = 0.18 and sd = 0.17) in the general population [22]. It indicated that 9 participants in each group would be sufficient to find a difference using a one-sample *t* test, with a power of .9 at a .05 significance level. Twenty adults with ASD and 19 controls were included in the study. Intelligence quotients were determined by the Wechsler Adult Intelligence Scale. No significant differences in age and intellectual quotient (IQ) were found between individuals with ASD and controls. Participants’ demographic characteristics are summarized in Table 1. All participants in the ASD group were recruited from the diagnostic clinic at Hôpital Rivière-des-Prairies, Montréal, Canada. All had been diagnosed by expert clinicians on the basis of DSM-IV (Diagnosis and Statistical Manual fourth edition) criteria, using standardized instruments (Autism Diagnostic Observation Schedule-Generic (ADOS-G) [23] and Autism Diagnostic Interview-Revised (ADI-R) [28]). Control participants were recruited through advertisements.

**Table 1:** Demographic characteristics of individuals with ASD and controls

	ASD	Controls	Statistics
Number	20	19	
Age	*M* = 25.2 (18–33), SD = 4.51	*M* = 22.79 (18–27), SD = 2.93	*W* = 134, *p* = .12
Intellectual quotient	*M* = 106 (86–129), SD = 11.8	*M* = 108 (87–124), SD = 11.3	*W* = 209, *p* = .59
Gender (male/female)	19/1	18/1	–
ADOS-G (*n* = 16)	*M* = 17.3 (10–25), SD = 3.86	–	–
ADI-R (*n* = 19)	*M* = 43.7 (34–55), SD = 5.58	–	–

*ADOS-G* Autism Diagnostic Observation Schedule-Generic [23], *ADI-R* Autism Diagnostic Interview-Revised [28]

### Procedure

**Fig. 1:**
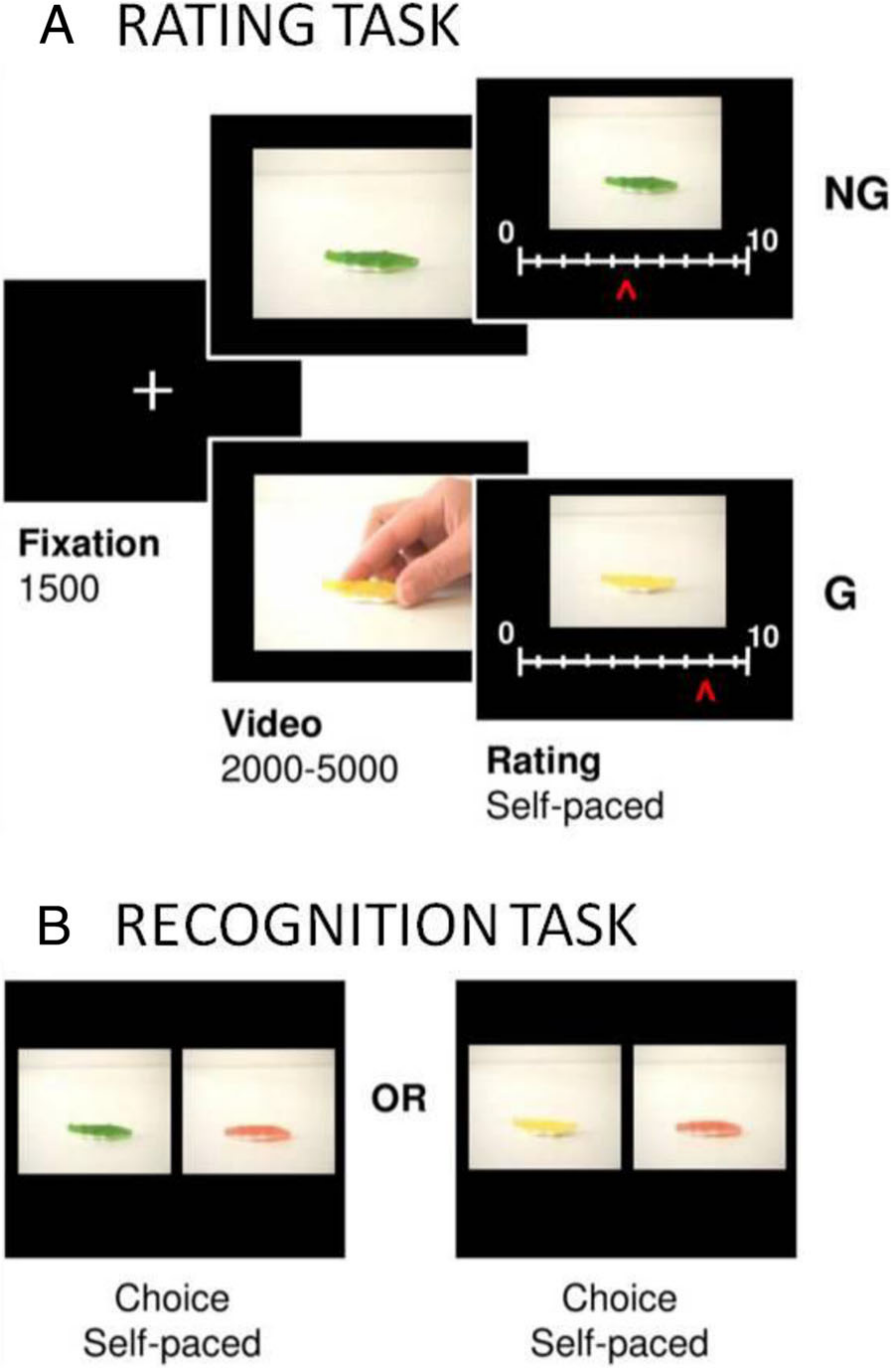
**a** The desirability-rating task. Successive screens displayed in one trial are shown from left to right with durations in milliseconds. Participants were instructed to rate “how much they would like to get/have the object.” Every trial of the task started with a fixation cross followed by the video. The desirability scale then appeared on the screen below the picture of the object to be rated (without human agent). The object was taken as the goal of an action in the G condition but not in the NG condition. Colors were counterbalanced at the group level. **b** The recognition task. Subjects had to select the “old” object, which meant the object that had been featured in the videos (either G or NG) shown during the rating task. Every choice contained one old and one “new” object. In the illustrated example, the correct answer would be *green* for the choice on the *left* and *yellow* for the choice on the *right*

All participants were tested in a quiet room, using a previously validated paradigm [22]. In the previous study, 120 different pairs of objects (e.g., food, toys, clothes, and tools) were selected to build the initial stimuli set. To make the task shorter for the present study, we selected the 60 pairs that showed the largest goal vs non-goal contrast on desirability in the previous study. Details about the stimuli can be found in Lebreton et al. [22]. Objects of two different colors were presented in short videos either as the goal of an action or not (G and NG conditions) (see Fig. 1). The face of the agent in the G videos was never shown, to avoid desirability being directly conveyed by facial expression. Also, a subset of NG videos included controls for the quantity of movement (with the object moving by itself) and for the presence of a human agent (not acting upon the object).

### Tasks

All tasks were programmed on a PC, using the Cogent 2000 (Wellcome Department of Imaging Neuroscience, London, UK) library of Matlab functions for presentation of stimuli. All participants took part in both a desirability-rating (test) task and a recognition (control) task. The desirability-rating task included 120 videos (60 object pairs), divided into two sessions. The two objects of a pair always appeared in the same session to limit the effects of temporal fluctuations and of session-wise rating scale anchors. Also, the presentation order of the different videos was randomized for each subject, with the constraint that the first and the second object of each pair should appear in the first and the second half of a session, respectively. To eliminate color preferences at the group level, color were counterbalanced between subjects.

In the rating task, participants were instructed to rate “how much they would like to have the object.” Every trial of the task started with a fixation cross displayed for 1.5 s and immediately followed by the video, which lasted between 2 and 5 s (see Fig. 1a). Next, the desirability scale appeared on the screen below the picture of the object to be rated (without a human agent). The scale was graduated from 0 (not desirable) to 10 (highly desirable). Participants could move the cursor by pressing a button with their right index finger to go left or with their right middle finger to go right. Rating was self-paced: subjects had to press a button with their left index finger to validate their response and proceed to the next trial. The initial cursor position on the scale was randomized to avoid confounding the ratings with the movements they involved. The total trial duration was almost 8 s on average (1500 ms of fixation + 3500 ms of video + 3300 ms of rating).

To allow interpretations of differences between groups in MD, we controlled for basic requirements of the task. First, to control for whether the subject paid attention to the objects, we administered a recognition task (see Fig. 1b), in which 60 pairs of pictures were presented. Each pair included an “old” picture, i.e., an object that the subject had seen during the rating task (in either a G or a NG video), and a “new” picture, i.e., the same object with a third and previously unseen color (which varied across object pairs). The order of the presentation was randomized for every subject. The two pictures of a pair were displayed side by side, following a 500-ms fixation cross. The relative position of the two pictures on the screen was also randomized. Subjects were asked to select the picture they had already seen (the “old” one). The task was self-paced.

MD may be associated with social motivation deficit in ASD, so we asked participants to complete questionnaires assessing social and physical anhedonia [5, 9, 13]. To control for possible confounding of motivation with depression, participants completed the Beck Depression Inventory [4]. As a measure of social cognition, participants also completed a test of social judgment on photographs [14].

## Analysis

Desirability ratings were converted to session-wise *z* scores. The first goal of the study was to assess whether MD (the difference in standardized desirability rating between G vs NG conditions) was lower in ASD than control participants. We used *t* tests to compare MD, ratings in the desire attribution task, and performance in the recognition task between groups (ASD vs controls). The data of the recognition task was missing for one participant due to an error in testing.

The second goal was to test whether MD was associated with clinical features of ASD. We looked for Pearson’s correlations between MD in the ASD group and the following variables: social and physical anhedonia scores and depression and social judgment scores. *p* values of correlations were not corrected for multiple testing since none did even reach the uncorrected threshold.

## Results

**Fig. 2:**
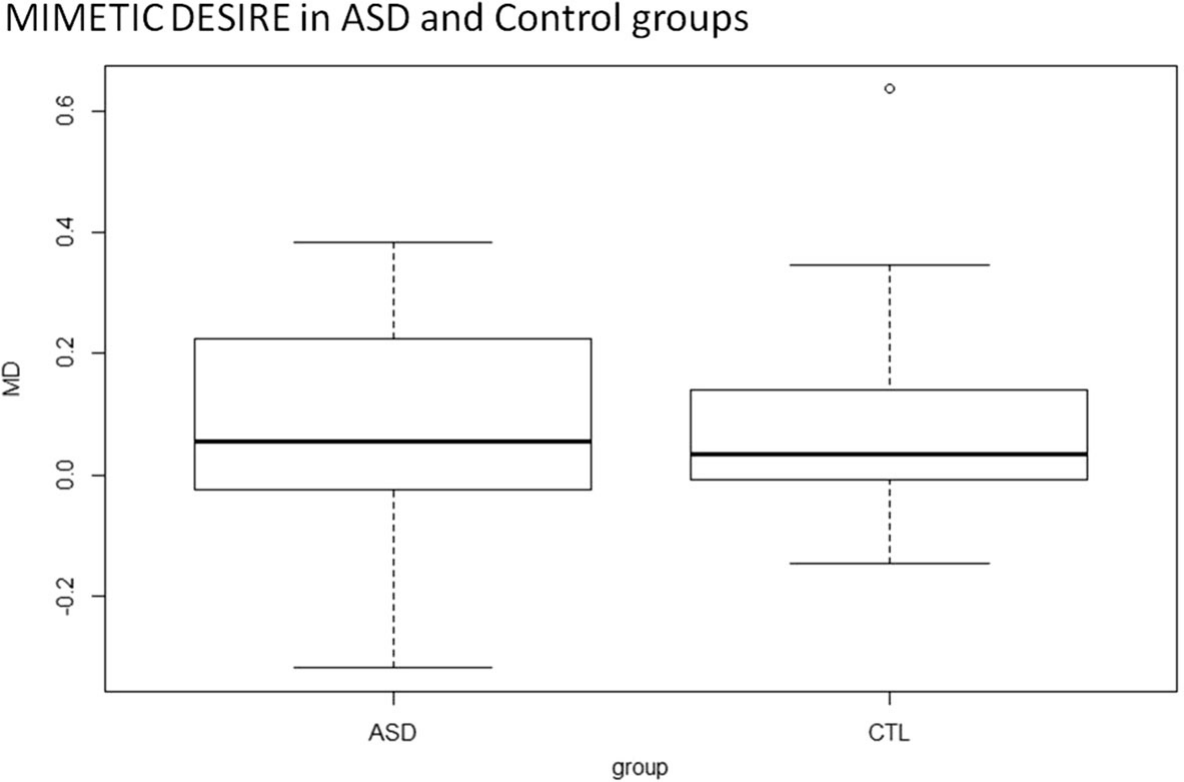
Comparison of MD in the ASD and control groups. Box plots show the minimum, first quartile, median, third quartile, and maximum of the MD effect (difference in desirability ratings between goal and non-goal objects) across individuals. MD was significantly positive in both groups, with no difference between groups

MD was present (i.e., MD >0) both in the ASD (one-sided *t* test *t*(19) = 2.08, *p* = .026, *d* = 0.46) and control (one-sided *t* test *t*(18) = 1.99, *p* = .031, *d* = 0.46) groups (Table 2). The main reason for using one-sided *t* tests to assess MD is that the analysis is confirmatory, since MD has already been found positive in five independent samples of participants [22]. A two-sample *t* test showed no difference between MD for the control and ASD groups (*t*(37) = −0.02, *p* = .98, *d* = 0.006) (Fig. 2). These results replicate the previous finding that MD is present in the general population, in a different sample in a different country (Canada) and also suggest that MD is similarly present in individuals with ASD. A post hoc power study based on MD in the two groups indicated that a sample of more than 20,000 participants per group would have been required to show a difference with a power of .7. This suggests that the observed absence of difference between the groups was not caused by the small sample size but reflects a true lack of difference between the populations.

**Table 2:** Results: between-group comparison of scores and correlations between MD and depression, anhedonia, and social judgment

	ASD	Controls	Comparison between groups	Correlation with mimetic desire
MD	*M* = 0.081, SD = 0.18	*M* = 0.082, SD = 0.18	*t*(37) = −0.02, *p =* .98, *d* = 0.006	–
Recognition	*M* = 0.83, SD = 0.09	*M* = 0.85, SD = 0.08	*t*(36) = −0.65, *p* = .52, *d* = 0.21	All: *r* = .14, *t*(36) = −0.83, *p =* .41 ASD group: *r* = .13, *t*(17) = 0.53, *p* = .60
Depression	*M* = 7.05, SD = 7.55	*M* = 3.68, SD = 3.6	*t*(37) = 1.76, *p =* .087, *d* = 0.55	All: *r* = .068, *t*(37) = 0.41, *p =* .68 ASD group: *r* = .21, *t*(18) = 0.90, *p* = .38
Social anhedonia	*M* = 15.7, SD = 7.57	*M* = 7.42, SD = 5.69	*t*(37) = 3.84, *d* = 1.06***	All: *r* = .03, *t*(37) = 0.18, *p =* .86 ASD group: *r* = .12, *t*(18) = 0.52, *p* = .61
Physical anhedonia	*M* = 20, SD = 9.72	*M* = 11.63, SD = 5.72	*t*(37) = 3.25, *d* = 0.93**	All: *r* = −.0031, *t*(37) = −0.019, *p =* .98 ASD group: *r* = .0066, *t*(18) = −0.03, *p* = .98
Social judgment	*M* = 0.71, SD = 0.11	*M* = 0.83, SD = 0.09	*t*(37) = 3.5, *d* = 0.98**	All: *r* = .15, *t*(37) = 0.93, *p =* .36 ASD group: *r* = .15, *t*(18) = 0.67, *p* = .51

**p < .01, ***p < .001

No between-group difference was found for recognition, suggesting that ASD and control participants paid equal attention to objects in the rating task. There were between-group differences in social judgment (higher performance by the control group), social and physical anhedonia (higher anhedonia in the ASD group), and a trend for higher depression scores in the ASD group; however, none of these factors were related to MD either in the entire sample (all *r* < .15, all *t*(37) < 1) or in the ASD subgroup (all *r* < .21, all *t*(18) < 1).

## Discussion

We found that individuals with an ASD are prone to MD to a similar extent as individuals in the controls. We found no link between MD and anhedonia or social judgment associated with ASD. These results contradict the intuitive idea that the preferences of individuals with ASD are less prone to social influence. They contribute to the understanding of social influence in autism.

There is a large body of literature on deficits in social influence in autism consistent with the notion that MD would be affected [10, 12]. A recent study [29] reported a failure to strategically use social cues so as to maximize payoff in situations of changing contingencies. However, some aspects of social influence have been found to be similar in individuals with and without autism. Individuals with autism share the stereotypes of their social group [7, 18, 19] and, like individuals without autism, can show better task performance in the presence of an observer [20]. Recent work indicates that both automatic and voluntary imitation of actions might be present [6] and even enhanced [30] in individuals with autism. Attention orienting by social cues appears to be preserved in such individuals (see [25] for a review, [27] for the exceptions). This suggests that at least some aspects of social influence are not abnormal in individuals with autism. Our investigation extends these observations by showing an absence of correlation between MD and any of the atypical social motivation and social cognition associated with autism.

Some basic mechanisms of social influence therefore seem to be intact in autism: the presence, goals, and representations of other people can influence a variety of behaviors, from gaze orientation to semantic associations and judgments about desirability. In contrast, differences have been described mostly in situations where control participants may strategically modulate their responses, notably to conform to social desirability using flattery or conformism: to confirm a statement (i.e., [8]), fawn (Chevallier et al. 2012), mask stereotypes [7], or appear more generous [20]. The current literature is thus consistent with the notion that individuals with ASD display less strategic behavior in social situations, but no basic deficit in social influence.

Our study has some limitations. One is that the study population was almost entirely male adults, due to availability for testing. This limits the generalization of the finding. A similar study with a sample of women and a larger age range would be useful. Indeed, it is plausible that the development of MD is delayed in, rather than absent from, individuals with ASD. Also, we have not investigated the underlying processes that may underpin MD. The absence of significant difference in desirability ratings does not preclude that individuals with ASD might have used different strategies at other levels (e.g., eye movements or brain activity), although such a speculation is not parsimonious.

## Conclusion

In conclusion, our study contributes to the understanding of social influence in ASD by showing that one of its core aspects, MD, is intact and not related to clinical or cognitive traits of ASD. Our findings suggest that the mechanisms at the neural level underlying MD (possibly the action of the mirror neuron system on the brain valuation system) are preserved in ASD. This weakens the notion that atypical interests in ASD stem from reduced social influence and therefore indirectly favors the idea that special interests might be selected by ASD individuals for their intrinsic properties [3, 24].

## Abbreviations

ADI-R: Autism Diagnostic Interview-Revised
ADOS-G: Autism Diagnostic Observation Schedule-Generic
ASD: Autism spectrum disorder
BVS: Brain valuation system
DSM-IV: Diagnosis and Statistical Manual fourth edition
G and NG: Goal and non-goal
IQ: Intellectual quotient
MD: Mimetic desire
MNS: Mirror Neuron System
